# Investigation of Phenolic Acids in Suspension Cultures of *Vitis vinifera* Stimulated with Indanoyl-Isoleucine, *N*-Linolenoyl-L-Glutamine, Malonyl Coenzyme A and Insect Saliva

**DOI:** 10.3390/metabo2010165

**Published:** 2012-02-15

**Authors:** Heidi Riedel, Divine N. Akumo, Nay Min Min Thaw Saw, Iryna Smetanska, Peter Neubauer

**Affiliations:** 1 Department of Food Technology and Food Chemistry, Methods of Food Biotechnology, Technische Universität Berlin, Königin- Luise-Str. 22, 14195 Berlin, Germany; Email: neminn@gmail.com (N.M.M.T.S.); iryna.m.smetanska@tu-berlin.de (I.S.); 2 Laboratory of Bioprocess Engineering, Department of Biotechnology, Technische Universität Berlin, Ackerstr. 71-76, D-13355 Berlin, Germany; Email: akumo2@yahoo.com (D.N.A.); peter.neubauer@tu-berlin.de (P.N.); 3 Department of Plant Food Processing, Agricultural Faculty, University of Applied Science Weihenstephan-Triesdorf, Steingruber Str 2, 91746 Weidenbach, Germany

**Keywords:** indanoyl-isoleucine, *N*-linolenoyl-L-glutamine, malonyl coenzyme A, insect saliva, *Vitis vinifera*, phenolic acids

## Abstract

*Vitis vinifera* c.v. Muscat de Frontignan (grape) contains various high valuable bioactive phenolic compounds with pharmaceutical properties and industrial interest which are not fully exploited. The focus of this investigation consists in testing the effects of various biological elicitors on a non-morphogenic callus suspension culture of *V. vinifera.* The investigated elicitors: Indanoyl-isoleucine (IN), *N*-linolenoyl-L-glutamine (LG), insect saliva (IS) and malonyl coenzyme A (MCoA) were aimed at mimicking the influence of environmental pathogens on plants in their natural habitats and at provoking exogenous induction of the phenylpropanoid pathway. The elicitors’ indanoyl-isoleucine (IN), *N*-linolenoyl-L-glutamine (LG) and insect saliva (IS), as well as malonyl coenzyme A (MCoA), were independently inoculated to stimulate the synthesis of phenylpropanoids. All of the enhancers positively increased the concentration of phenolic compounds in grape cells. The highest concentration of phenolic acids was detected after 2 h for MCoA, after 48 h for IN and after 24 h for LG and IS respectively. At the maximum production time, treated grape cells had a 3.5-fold (MCoA), 1.6-fold (IN) and 1.5-fold (IS) higher phenolic acid content compared to the corresponding control samples. The HPLC results of grape cells showed two major resveratrol derivatives: 3-*O*-Glucosyl-resveratrol and 4-(3,5-dihydroxyphenyl)-phenol. Their influences of the different elicitors, time of harvest and biomass concentration (*p* < 0.0001) were statistically significant on the synthesis of phenolic compounds. The induction with MCoA was found to demonstrate the highest statistical effect corresponding to the strongest stress response within the phenylpropanoid pathway in grape cells.

## 1. Introduction

Grapevine (*Vitis vinifera*) is one of the major fruit crops worldwide but is also very susceptible to microbial and fungal attacks [1]. Wine berries are a valuable rich source of bioactive secondary metabolites especially for polyphenolics such as phenolic acids and catechins [2] as well as for anthocyanins like cyanidin 3-glucoside, peonidin 3-glucoside, malvidin 3-glucoside, cyanidin 3-*p*-coumaroyl glucoside, peonidin 3-*p*-coumaroyl glucoside and malvidin 3-*p*-coumaroyl glucoside [3,4]. The food and biopharmaceutical industry is highly interested in plant derived polyphenolics because of their taste, color, flavor, their role in food preservation [5] as well as for their antioxidant properties for different preventive role against diseases such as cancer, cardiovascular and neurodegenerative diseases [6,7]. In response to stress, plants activate their transcriptional protective mechanisms at the molecular level: (i) by an increase of antioxidant enzymes and metabolites; (ii) by induction of protection-related secondary metabolite genes; and (iii) by the opening of ion channels and modification of protein phosphorylation status [6,8]. After attacking and wounding, plants emit toxins or volatile chemicals in response to herbivores [9]. The composition of the saliva is a mixture of various enzymes, especially oxidase, such as cytochrome P 450 oxidases, which are responsible for the reduction of the effectiveness of many toxic secondary metabolites in plants [10,11] and activate defense mechanisms in plant cells. Most of these nonessential substances are derived from the isoprenoid, phenylpropanoid, alkaloid or fatty acid/polyketide pathways [12]. The present study focuses on the production of valuable bioactive phenolic compounds which could be of medicinal use. A novel biotechnological approach to traditional plants cultivation is the use of *in vitro* cultures as a tool for the synthesis of bioactive metabolites. Through *in vitro* cell culture, the accumulation of the target compounds can be enhanced by stimulation with different elicitors [13]. In this investigation, different biological elicitors (*N*-linolenoyl-L-glutamine, indanoyl-isoleucine, insect saliva) and malonyl coenzyme A were used to stimulate the synthesis of phenolic acids in grape cells. Indanoyl isoleucines (IN) conjugates are synthetic substances which mimics signals from the octadecanoid pathway which occur in plants for resistance properties against insect attacks [14]. IN was already earlier applied as activator and regulator to influence the gene expression and production of secondary metabolites such as phenolic acid synthesis. Coronatine is a structural analogue of IN and was isolated functionally analyzed from *Pseudomonas syringae* pathovars. It functions as a bacterial phytotoxin with similar biological activities, such as jasmonates. However, the activity of coronatine was appr. 100–10,000 times higher compared to jasmonic acid [15]. Berrin, found out that a cell suspension culture of *Linum nodiflorum* treated with indanoyl-isoleucine accumulated 10-fold of 6-methoxypodophyllotoxin (MPTOX) [16] compared to untreated cells. Another biological stimulant for secondary metabolites is the oral section of insects with a high variety of signaling compounds which cause the emission of volatiles by plants [17]. *N*-linolenoyl-L-glutamine (LG) is an important component of insect saliva (tobacco hornworm) and is responsible for the majority of elicitor activity in plants [18,19]. *N*-linolenoyl-L-glutamine LG) has a similar chemical structure as other elicitors including volicitin, which was the first fatty acid amide elicitor identified in the caterpillars of *Manduca sexta* [20]. *N*-linolenoyl-L-glutamine is derived from the fatty acid linolenic acid and is the precursor of jasmonic acid, synthesized by the octadecanoid pathway. Enzymes generally function as catalyst in metabolic pathways, such as in the synthesis of natural metabolites, and can also be used as enhancer for valuable compounds. Malonyl Coenzyme A is one of the most important intermediate with regulatory functions in the fatty acid and polyketide synthesis pathways. It provokes the production of stilbenes like Resveratrol, which is one of the major compounds in grapes [21]. It is mainly due to the high antioxidative potential of Resveratrol that grapes are claimed to have health-promoting properties. For this reason MCoA was used as stimulant in *in vitro* grape cultures. Insect Saliva (IS) of the tobacco hornworm was used as one of the elicitors to mimic herbivore attack because it is reported to stimulate the production of direct or indirect defense mechanisms [22]. Stimulation includes many levels, such as genome organization, gene expression control and consequent induction of whole metabolic pathways, as well as synthesis of specific metabolites [18,23,24]. The present study was aimed at investigating the effect of the above-mentioned biological elicitors on a grape suspension culture and their influence on the synthesis of health-promoting compounds related to the phenylpropanoid pathway.

## 2. Results and Discussion

The aim of this study was to investigate and enhance the concentration of bioactive phenolic compounds in the suspension culture of *V. vinifera* after treatment with biological elicitors. Grape cells were stimulated with *N*-linolenoyl-L-glutamine (LG), indanoyl-isoleucine (IN), malonyl coenzyme A (MCoA) and insect saliva (IS), and their resulting impact on cell growth, production of phenolic acids and other influencing factors was investigated.

### 2.1. Growth Kinetics, Phenolic Acids of the Culture and HPLC Analysis

**Figure 1:**
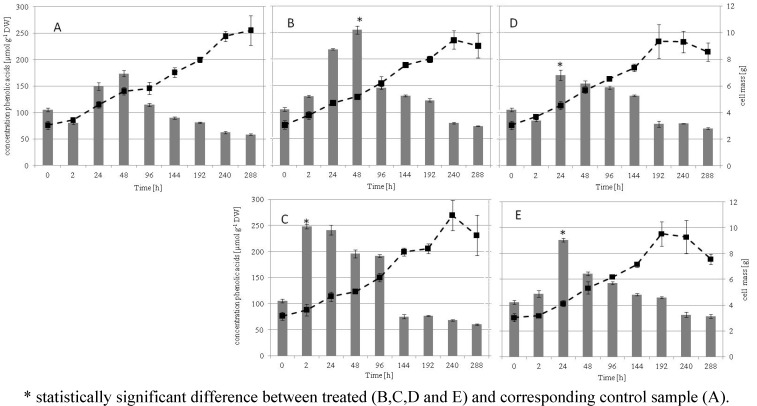
Multicomparison between growth and accumulation of phenolic acids in grape cells. The bars represent the concentration of phenolic acids and the lines represent cell mass. (**A**) control, (**B**) IN, (**C**) MCoA, (D) LG, (**E**) IS.

The growth kinetics and the phenolic acid production of grape suspension cell culture after treatment with *N*-linolenoyl-L-glutamine (LG), indanoyl-isoleucine (IN), insect saliva (IS), malonyl coenzyme A (MCoA) against the control grape cells and of the untreated cells are shown in Figure 1.

The biomass of all cultures (treated and untreated) showed similar dynamically linear growth up to 240 h, followed by a stationary phase for all treated cultures, whereas growth proceeded in the control sample even after 288 h. The treated cultures started to propagate from time zero until they reached their maximal cell densities at 192 h (LG and IS) and 240 h (IN and MCoA) after treatment respectively before dropping. The maximum biomass of all cultures was approximately 10 g at 240 h. It is obvious that elicitation had no immediate inhibitory effects on the growth of treated grape cell cultures compared to that of the control samples. These results suggest that in situations in which biomass is the goal of production, no treatment is needed. Nevertheless, treated grape cells were found to trigger many metabolic pathways for the synthesis of secondary metabolites of economic interest. There was a rapid accumulation of phenolic acids in the cultures treated with MCoA and IN reaching its maximal after 2 h and 48 h respectively. The highest concentration of phenolic acids after treatment with LG and IS was detected after 24 h. The highest phenolic acid content per cell unit was 3.5-fold (MCoA: 2 h); 1.6-fold (IN: 48 h) and 1.5-fold (IS: 24 h) at the distinct time where the highest concentration was detected, compared to the concentration at the same time of the corresponding control sample without elicitation. Estimates of phenolic acid concentration per cell unit were as follows; grape cells treated with MCoA was about 1,000 µmol after 2 h compared to control with about 300 µmol. Interestingly, the concentration of phenolic acids after 2 days after IN treatment per cell unit was 1,250 µmol whereas the amount in untreated cells was about 1,020 µmol. This is similar to the suspension cells treated with LG (24 h). In addition, in this case, their phenolic acid content was only slightly higher than that of the control. Based on multiple comparison tests, there were strong statistically significant differences between the treated grape cells treated with MCoA, (LG and IS) and IN after 2, 24 and 48 h and their corresponding control counterparts (*p* < 0.0001). The effect of the biological elicitors to enhance the synthesis of phenolic acid within the first 48 h was MCoA > IN > IS > LG. MCoA showed the fastest response. However, this strong enhancement in phenolic acid content by the different biological stimulants (MCoA, IN, IS and LG) is gradually lost over time because of homeostatic balance within the cells. These results suggest that although all treatments did enhance phenolic acid synthesis; for a rapid harvest of high yield phenolic acid, it will be better to treat grape cells with malonyl coenzyme A.

### 2.2. Chemical Analysis of *in Vitro* Grape Cells with HPLC

**Figure 2:**
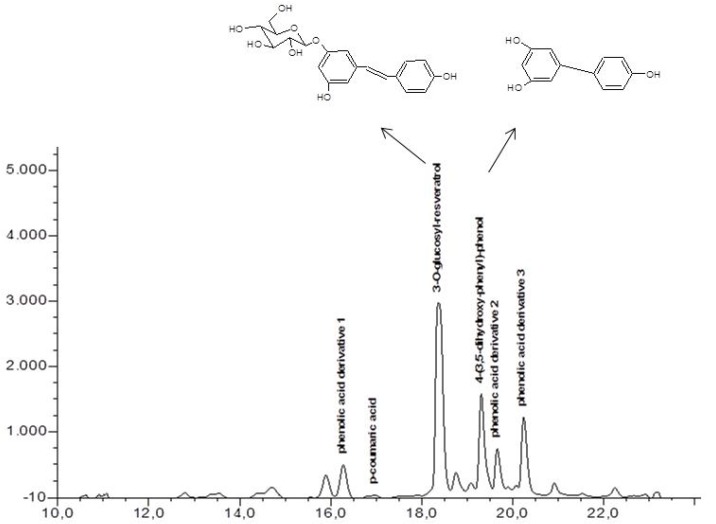
Chemical structures of major resveratrol derivatives identified with the HPLC.

Figure 2 is an HPLC chromatogram from extracts of suspension cell cultures (*V. vinifera)* of untreated samples. Two major phenolic compounds; 3-*O*-glucosyl-resveratrol and 4-(3,5-dihydroxyphenyl)-phenol (compound **5** and **6**) as well as the internal standard *p-* coumaric acid were identified. The HPLC chromatogram shows the identified phenolic compounds at their respective retention time (min). The reproducibility of phenolic compounds was very efficient with high correlation coefficients (R^2^ = 0.9998) for the different linear equations. The chemical structures of the two very important resveratrol derivatives identified in the grape cell cultures; 3-*O*-glucosyl-resveratrol and 4-(3,5-dihydroxyphenyl)-phenol are shown in Figure 2.

### 2.3. Effects on the pH and Conductivity

**Figure 3:**
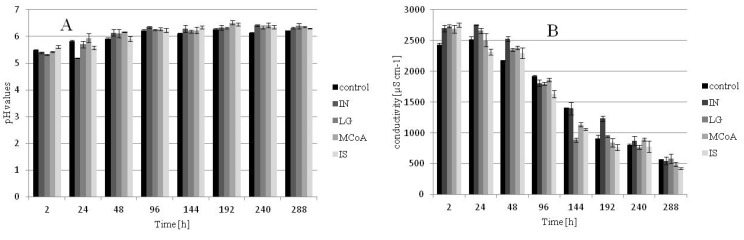
pH values (**A**) and conductivity (**B**) measured of the medium.

The influence of pH and conductivity the grape cells and the culture medium were investigated and are demonstrated in Figure 3A,B. No statistically significant differences could be detected between the pH and conductivity of the different cultures, suggesting that both parameters did not directly influence the synthesis of phenolic acids. The estimated pH values for the treated and untreated samples showed a similar trend which was an increase over time from pH 5.4 to pH 6.2, whereas the conductivity in the medium decreased over the time. Furthermore there was no inhibitory effect on growth after elicitation. In addition, changes in pH and conductivity were closely correlated with the increase in biomass production over time.

### 2.4. Influence of Experimental Parameters on Phenolic Acid Synthesis

The effects of the experimental parameters (treatment, time, pH, conductivity, dry/fresh weight) on the synthesis of phenolic acid were statistically tested with ANOVA. The type of treatment, time of harvest, dry / fresh weight and the interaction between time of harvest and treatment were found to all influence phenolic acid content as shown in Table 1.

**Table 1:** ANOVA test for experimental parameters influencing phenolic acids content.

Parameter	DF	Mean Square	F Value	*p*-value
Treatment	5	7453.09	146.06	<0.0001
Time (harvesting time)	7	35004.10	687.60	<0.0001
pH	1	564.18	11.06	0.0013
Fresh weight	1	223.30	4.38	0.0396
Treatment * Time	28	2955.58	57.92	<0.0001

DF = degree of freedom.

The main objective was to investigate the effects of different biological elicitors (indanoyl-isoleucine, *N*-linolenoyl-L-glutamine, malonyl coenzyme A and insect saliva) on the synthesis of phenolic acids in *in vitro* cell cultures of *V. vinifera*. This is one of the first studies in which different elicitors of biological origin were used in grape suspension cultures to stimulate the phenylpropanoid pathway and enhance bioactive phenolic compounds. After analyzing the treated and untreated samples on HPLC, high amounts of phenolic compounds with two major resveratrol derivates (3-*O*-glucosyl-resveratrol and 4-(3,5-dihydroxyphenyl)-phenol) were present in very high concentrations. The synthesis and accumulation of resveratrol derivates corresponds to the beginning of exponential cell growth phase in plant cells. These results were similar to previous findings by Waffo *et al*. [25]; who hypothesized that the production of stilbenes is coupled to cell growth [25]. In other studies, similar findings have been demonstrated with anthocyanins and condensed tannins [4,26]. Furthermore, the accumulation of resveratrol in grape cells has been previously reported in other studies [27,28,29]. Resveratrol is one of the major compounds found in grape and is of high pharmaceutical importance because of its cardioprotective and anticarcinogenic activity [30]. In this study the highest amounts of phenolic acids after treatment of grape cells with the key metabolite MCoA were detected within 2 h to increase up to 250 µmol g^−1^ DW. Malonyl Coenzyme A plays an important role in the biosynthetic phenylpropanoid pathway most especially for the synthesis of stilbenes [31]. For the synthesis of resveratrol, three molecules of MCoA and one molecule of *p*- coumaroyl CoA are needed [32]. Malonyl-CoA is the precursor for the fatty acid synthesis and the core of the building blocks to the synthesis of; phytoalexins, flavonoids and many other malonylated compounds [33]. Phenolic compounds are known as very important phytoalexins accumulated in plants because of their biological activities against various pathogens and environmental factors [34,35]. Among the biological elicitors used in this study, the treatment of grape cells with indanoyl isoleucine also stimulated the production of phenolic acids dramatically after 48 h to a level of 252 µmol g^−1^ DW. A similar effect was seen for previous investigation showing the influence of jasmonic acid on the biosynthesis of phenolic acids whereby the maximum production was achieved also 2 days after stimulation. Berim *et al*. also investigated the influence of the synthetic elicitor indanyl isoleucine on the lignin production in a suspension culture of *Linum nodiflorum* [16]. After he treated his cell cultures with IN, the production of 6-methoxypodophyllotoxin (MPTOX) was enhanced and additional lignans accumulated in the treated cultures [16]. Until now there have been no experiments using elicitation of plant cell cultures with insect derived substances such as LG and insect saliva for the stimulation of phenolic biosynthesis. *N*-linolenoyl-L-glutamine (LG) which is an amide of linolenic acid and an analog to various elicitor activities including volicitin [18,36,37]. It was the first fatty acid amide elicitor identified in caterpillars of *Manduca sexta* [20]. This substance was found in oral secretions of caterpillars; plants respond to attacks with a high release of volatile compounds as a defense mechanism [36]. For *N*-linolenoyl-L-glutamine (LG) which is an amide of linolenic acid in our cultures the highest amount of phenolics were observed 24 h after treatment with LG raising the concentration of phenolic acids to 270 µmol g^−1^ DW. However, there were no significant differences in phenolic acid contents between grape cells treated with LG and untreated samples after the 96 h. LG belongs to the class of biotic elicitors which are produced by herbivore pests and are able to rapidly activate various plant chemical defense mechanisms when exposed to plant tissue. It is an insect‑derived volicitin and plays a key role as activator in signal volatiles [38]. The grape cells were also stimulated with insect saliva derived from *Manduca sexta* (tobacco hornworm) which contains many different molecules to serve as activators in plant defense mechanisms [22]. Insect saliva (IS) affected phenolic acid synthesis within 24 h and provided a 1.5-fold increase compared to the corresponding control sample. The results show that the biological stimulants had no inhibitory effects on cell growth of treated cells. Some earlier studies demonstrate that IN caused a decrease of cell growth in a *Linum nodiflorum* suspension culture [16]. Different exogenous parameters such as concentration of elicitor, time of application, plant species and cultivation conditions can influence the growth. The synthesis of secondary compounds has mostly negative effects on cell growth [39], but in this experiment we did not observe a significant reduction of biomass formation.

## 3. Materials and methods

### 3.1. Cultivation and Maintenance of Vitis vinifera c.v. Muscat de Frontignan

The suspension culture of *V. vinifera* was established by Francois Cormier (Food Research and Development Centre, Agriculture Canada), and has been under cultivation for 15 years in the Department of Food Biotechnology and Process Engineering at the TU Berlin. The grape plant cells were cultivated on B5 basal medium (Gamborg B5 Medium B5VIT, Duchefa B.V., The Netherlands) supplemented with 0.1 mg/L 1-Naphthaleneacetic acid (-NAA), 0.2 mg/L kinetin, 0.25 g/L casein hydrolysate (Merck, Darmstadt), 3% sucrose and 0.8% agar. *V. vinifera* plant cells with deep red color were selected once in a while to assure homogenic conditions. Erlenmeyer flasks were kept at 25 °C in 24 h photoperiods under a fluorescent lamp (approx. 3,000 lux) on an orbital shaker at 100 rpm. The plant cells were propagated into fresh medium under sterile conditions for every 14 days.

### 3.2. Chemicals

The solvents and Malonyl Coenzyme A used in this experiment were analytical graded and were ordered from Sigma (St. Louis, MO, USA). Meanwhile, *N*-linolenoyl-L-glutamine and indanoyl-isoleucine were kindly provided by Prof. Dr. W. Boland of Max- Planck- Institute of Chemical Ecology Jena. The insect saliva of the tobacco hornworm *Manduca sexta* was provided by Prof. A. Steppuhn of the Free University Berlin.

### 3.3. Culture Preparation

The experiment was performed in 100 mL Erlenmeyer flasks containing 25 mL of B5 basal medium. After sterilization at 121 °C for 25 min, 4 g fresh weight plant cells of *V. vinifera* were inoculated in to each flask. The flasks (triplicate) were harvested after 2, 24, 48, 96, 144, 192, 240 and 288 h respectively after treatment with *N*-linolenoyl-L-glutamine, indanoyl-isoleucine, malonyl coenzyme A and insect saliva. Meanwhile the untreated samples (control) were simultaneously harvested. Furthermore, the flasks from pool (0 h) were collected and analyzed.

### 3.4. Preparation and Treatment with Elicitors

In 100 ml Erlenmeyer flasks containing 25 mL Vitis media each, 4 g of fresh weight plant cells from *V. vinifera* (without using vacuum) were inoculated into each flask. To enhance the synthesis of phenolic compounds, plant cells were stimulated with: *N*-linolenoyl-L-glutamine (LG), indanoyl-isoleucine (IN), Malonyl Coenzyme A (MCoA) and insect saliva (IS). Prior to preliminary concentration testing, the elicitors were added to the media at day 0 and standardized to a concentration of 0.2 mg per 25 mL. The stock solutions of each substance were sterilized by filtration (0.22 µm). The experiment was made up of triplicates of every treatment (LG, IN, MCoA and IS) and control (no treatment). Samples from each triplicate flask with and without treatments were harvested after 2, 24, 48, 96, 144, 192, 240 and 288 h for the determination of fresh and dry weight, pH, conductivity, phenolic compounds (phenolic acids, anthocyanin) after stimulation and also from pool (0 h).

### 3.6. Estimation of Experimental Parameters from Plant Cells and Medium

The following parameters were measured from each sample; pH, conductivity, fresh and dry weight and phenolic compounds. The pH meter (CG811; Schott Geräte GmbH, Hofheim, Germany) and conductivity meter (WTW LF 323; Weilheim, Germany) were used to estimate the pH and conductivity of metabolic end products and the nutrient contents in the medium. The pH and conductivity of every sample were measured within a time lapse of 30 s to stabilize both parameters at room temperature. The plant cells were filtered using suction filter (SARSTEDT, Germany) in a vacuum for one minute followed by weighting. One-gram of fresh plant material was dried in a prepared aluminum box and kept at 105 °C in an oven for 24 h. After the drying process, the samples were transferred for one hour in to an exsiccator. Thereafter, the dry weights were measured and the water content was calculated. Plant cells of *V. vinifera* were harvested using vacuum filtration flask. At each day of harvest, the fresh and dry weights, pH and conductivity were estimated and the chemical components were analyzed. The harvested plant cells for the phenolic acid extraction were immediately flash frozen in liquid nitrogen and transferred for the freeze-drying process (lyophilization).

### 3.7. Chemical Analysis of Phenolic Acids with HPLC

For chemical analysis, about 40 mg powdered callus samples (freeze-dried) were extracted within 15min using 750 µL of 70% methanol (v/v; pH 4; 0.1% phosphoric acid) containing 40 µL of the internal standard *p*-coumaric acid (3 mmol) in an iced ultrasonic water bath. All samples were centrifuged at 4,500 rpm (2,150 × g) and 4 °C for 5 min. The supernatants were collected in new tubes and the pellets were re-extracted with 500 µL of 70% methanol (twice). After extraction, aliquots of the samples were collected and the solvent was completely removed using a rotary evaporator (Speed Vac, SC 110) under vacuum at room temperature (25 °C). The residues were filtered using centrifuge tubes (SpinX) and the extracts were dissolved with 40% acetonitrile to reach the 1 mL mark. The chromatography was performed using a Dionex Summit P680A HPLC system with an ASI-100 auto sampler and a PDA-100 photodiode array detector. Hydroxycinnamic acid derivatives were separated on a narrow bore Acclaim PA C16-column (150 × 2.1 mm, 3 µm, Dionex) with an injection volume of 40 µL and a temperature of the column oven 35 °C. The eluent flow rate used was 0.4 ml min^−1^. A 39min gradient program was used with 1% (v/v) phosphoric acid in ultrapure water (eluent A) and 40% (v/v) acetonitrile in ultrapure water (eluent B) as follows: 1 min 0.5% (v/v) B, a gradient from 0–40% (v/v) B for 9 min, with a 2 min hold, a gradient from 40–80% (v/v) B for 6 min, with a 2 min hold, gradient from 80–99% (v/v) B for 4 min, a gradient from 99–100% (v/v) B for 6 min, a gradient from 100–0.5% (v/v) B for 4 min and a final step at 0.5% B for 5 min. Peaks were monitored at 290, 330 and 254 nm respectively. The phenolic acid quantity was calculated from HPLC peak areas at 290 nm. The retention times in the HPLC for the experiments were 12.13 min for vanillic acid, 12.72 min for chlorogenic acid, 13.29 min for caffeic acid, 15.98 min for the internal standard *p*-coumaric acid and 21.59 min for cinnamic acid. For the identification of unknown phenolic compounds, a semi-quantitative analysis was performed using HPLC coupled with mass spectrometric detection (LC/MS). Chromatography was performed using a Finnigan MAT95S (EI samples) and Orbitrap LTQ XL (Thermo Scientific) for the ESI samples. The spray voltage of the electro-spray ionization was 5 kV with the source temperature 275 °C. The solvent was a mixture of methanol with 0.1% formic acid and at a flow rate of 200 µL·min^−1^. The flow rate of the syringe pump was 5 µL·min^−1^. Gradient elution solvent A was water mixed with 0.1% formic acid and solvent B was methanol with 0.1% formic acid. The flow rate in the HPLC gradient program was 1 mL·min^−1^ and the elution started at time 0min with 95% of solvent A and 5% of solvent B. After 25 min, the solvent composition was 0% and 100% for solvents A and B respectively which remain the same until the 38 min. At the terminal phase, between 38.01 min and 40 min, the solvent composition was 95% of solvent A and 5% of solvent B.

### 3.8. Statistical Analysis

The data sets were made up of triplicates for every trial per treatment and control group across different time of harvest and are reported as least square means (LSM) ± standard deviation (SD). The general linear model (GLM) of the statistical package SAS (2003) for Windows, version 9.1 (SAS Institute, Cary, NC, USA) including all significant factors was used for data analyses. The experimental data were subjected to analysis of variance (ANOVA) followed by multiple comparison tests between estimated LSMs for phenolic acid content between and within treatment trials post Tukey’s Kramer test. The F-test was used to assess statistical significance of effects at 95% confidence interval. The level of statistical significance was assigned at *p*-values ≤ 0.05 for all statistical analyses. The following general linear model was used in investigating the effects of different factors and the differences between and within treatments on phenolic acid contents (Model 1).


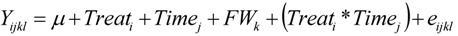
Model 1

where;

Y_ijkl_ = Phenolic acid content,

µ = Overall mean of phenolic acid content,

Treat_i_ = fixed effect of Treatment (i = IN, LG, IS, MCoA and control),

Time_j_ = fixed effect of harvesting time in hours (j = 2, 24, 48, 96, 144, 192, 240 and 280),

FW_k_ = fixed effect of fresh weight (k = sample),

Treat_i_*Time_j_ = interaction between treatment and time of harvest (i = treatment, j = time)

e_ijkl_ = residual error.

## 4. Conclusions

This study showed and confirmed many phenolic metabolites in a grape suspension culture such as stilbenes, phenolic acid and anthocyanins, to name just a few. Treatment with IN, LG, MCoA or IS did not provide any significant inhibitory effect on the cell growth. The stimulation with biological substances such as IN, saliva and MCoA improved the biosynthesis of phenolic compounds and promising higher yields of bioactive metabolites. The rapid effect of these biological stimulants on the amounts of phenolic substances may be of high pharmaceutical importance as well as economic value because of the high exploitation rate of secondary metabolites within a very short time lapse. Although all treatments positively influenced the synthesis of phenolic acid and biomass, it is advisable to use them for phenolic acid extraction rather than biomass. The major reason is the inhibitory effect of the stimulants on cell propagation after some time, whereas indefinite growth is achieved with untreated grape cells. Nevertheless, MCoA was the preferred stimulant with the highest yield in phenolic acid within just 2 h of treatment. Furthermore, MCoA is an important natural regulator and metabolite in the biosynthesis of phenolic compounds. It remains of interest to evaluate in future studies whether the effect of MCoA is by its regulatory role or as a direct substrate. Naturally, plants have to activate their defense mechanisms by producing signaling molecules within a short time for survival. This explains why different biological elicitors used in this study serve as excellent stimulants to plant *in vitro* cultures of *V. vinifera*. Although MCoA directly may be too expensive for use in a production process, our results may provide ideas for genetic modifications or metabolic treatments to obtain a similar effect, also with cheaper compounds.
